# Preoperative fasting glucose value can predict acute kidney injury in non-cardiac surgical patients without diabetes but not in patients with diabetes

**DOI:** 10.1186/s13741-024-00398-4

**Published:** 2024-05-13

**Authors:** Qianyun Pang, Yumei Feng, Yajun Yang, Hongliang Liu

**Affiliations:** Department of Anesthesiology, Chongqing University Cancer Hospital, Hanyu Road 181, Shapingba District, Chongqing, 400030 People’s Republic of China

## Abstract

**Background:**

Postoperative acute kidney injury (AKI) is a common and costly complication after non-cardiac surgery. Patients with or without diabetes could develop hyperglycemia before surgery, and preoperative hyperglycemia was closely associated with postoperative poor outcomes, but the association between preoperative fasting blood glucose level and postoperative AKI is still unclear.

**Methods:**

Data from patients undergoing non-cardiac surgery in Chongqing University Cancer Hospital from January 1, 2017, to May 31, 2023, were collected, preoperative glucose value and perioperative variables were extracted, the primary exposure of interest was preoperative glucose value, and the outcome was postoperative AKI.

**Results:**

Data from 39,986 patients were included in the final analysis, 741(1.9%) patients developed AKI, 134(5.6%) in the cohort with DM, and 607(1.6%) in the cohort without DM(OR 1.312, 95% CI 1.028–1.675, *P* = 0.029). A significant non-linear association between preoperative glucose and AKI exists in the cohort without DM after covariable adjustment (*P* = 0.000), and every 1 mmol/L increment of preoperative glucose level increased OR by 15% (adjusted OR 1.150, 95% CI 1.078–1.227, *P* = 0.000), the optimal cut-point of preoperative fasting glucose level to predict AKI was 5.39 mmol/L (adjusted OR 1.802, 95%CI 1.513–2.146, *P* = 0.000). However, in the cohort with DM, the relation between preoperative glucose and postoperative AKI was not significant after adjusting by covariables (*P* = 0.437). No significance exists between both cohorts in the risk of AKI over the range of preoperative glucose values.

**Conclusion:**

A preoperative fasting glucose value of 5.39 mmol/L can predict postoperative acute kidney injury after non-cardiac surgery in patients without diagnosed diabetes, but it is not related to AKI in patients with the diagnosis.

**Supplementary Information:**

The online version contains supplementary material available at 10.1186/s13741-024-00398-4.

## Introduction

Postoperative acute kidney injury(AKI) is a common and costly complication after non-cardiac surgery, and it is closely associated with an increase in other postoperative complications, prolonged hospital stay, development of chronic kidney injury, and short- or long-term mortality (Meersch et al. 2017; Grams et al. 2016; Kork et al. 2015). A recent Animal study reported that acute hyperglycemia could cause obvious tubular morphological and functional injuries in rats (Wang et al. 2020). Patients with or without diabetes could develop hyperglycemia before surgery due to poor control of blood glucose or preoperative stress from anxiety, trauma, and others, and preoperative hyperglycemia was closely associated with postoperative poor outcomes (Chiang et al. 2021; Zhang et al. 2022), but the relation between preoperative fasting blood glucose level and postoperative AKI is unclear till now.

The guidelines recommend that the perioperative fasting blood glucose should be controlled below 10 mmol/L or a minimum between 4.4 and 8 mmol/L as the optimal range for elective surgeries to avoid harm from hypoglycemia or hyperglycemia (Dhatariya et al. 2012; Cosson et al. 2018; Association of Anaesthetists of Great Britain and Ireland 2015). Till now, there is no unique target for preoperative blood glucose control in patients with or without DM, and to our knowledge, the optimal level of preoperative fasting blood glucose to minimize postoperative AKI is unclear. In this retrospective cohort study, we investigated the potential association between preoperative fasting blood glucose level and postoperative AKI in non-cardiac surgery, identified the cut-off value to predict postoperative AKI, and observed the difference in the risk of AKI development between the cohorts with and without DM.

## Methods

This study was approved by the Ethics Committee of Chongqing University Cancer Hospital (No. CZLS2024020-A), as it was a retrospective study and the data was anonymous, the informed consents from patients were waived.

The participants were those who underwent non-cardiac surgery under general anesthesia in Chongqing University Cancer Hospital from January 2018 to May 2023. The inclusion criteria were age > 18 years, elective non-cardiac surgery, surgical duration > 1 h, general anesthesia, and hospitalization for at least 2 days after surgery. The exclusion criteria were end-stage kidney disease, not enough data to determine postoperative AKI and data on preoperative glucose was missing. For those who underwent multiple surgeries during the study period, the data were obtained from the initial surgery. The patients consist of two cohorts, the cohort with DM (known DM, referring to the population with a history of DM or long-term use of hypoglycemic drugs or insulin) and the cohort without DM (no known DM).

The data of surgical patients who met the inclusion criteria were collected through the electronic medical record system of Chongqing University Cancer Hospital. The characteristics of patients included age, sex, ASA physical status (ASA PS), body mass index (BMI), smoking, comorbidities (hypertension, coronary artery disease, heart failure, COPD, cancer, stroke, arrythmia, renal disease, liver disease, thyroid disease), long-term medication (NSAIDS, steroid, antihypotensive agents, hypoglycemic agents, insulin), preoperative laboratory tests (hemoglobin, albumin, fasting blood glucose, creatinine), intraoperative data (body temperature, blood pressure, fluid infusion rate, bleeding, transfusion, hydroxyethyl starch, noradrenaline, any episode of hypotension, NSAIDs), and postoperative creatinin levels within 7 days. The blood sample was withdrawn from a peripheral vein on the morning before oral intake within 72 h before surgery, and the preoperative serum glucose level was measured at the central laboratory of Chongqing University Cancer Hospital. If there were multiple fasting blood glucose values before surgery, the latest one to the surgery was used for data analysis.

The primary outcome was postoperative AKI. AKI was defined as an increase in serum creatinine of ≥ 26.5 μmol/L within 48 h or ≥ 1.5 times the baseline value within 7 days after surgery according to KDIGO(Kidney Disease Improving Global Outcomes) criteria (Kellum et al. 2012).

### Statistical analysis

The categorized variables were presented as *n* (%), and the continuous variables were presented as median (interquartile range) in all patients, DM cohort, and non-DM cohort. The distribution of preoperative fasting glucose values in 1.0 mmol/L increments was plotted in frequency histograms for patients with and without DM.

When the data of preoperative glucose or intraoperative blood pressure or for postoperative AKI diagnosis were missing, the data of the patient were excluded from the final analysis. Other variables (e.g., the patients’ characteristics or laboratory results) had missing values of < 5%, and we imputed the missing data with the median of each cohort and incorporated all the data for the subsequent multivariable analysis.

The restrictive cubed spline model was used to determine the potential associations between preoperative fasting glucose level and postoperative AKI in cohorts with DM and without DM, and logistic regression analysis was used to calculate the adjusted odds ratio (OR) for all the covariables listed in Table 1. The adjusted OR and 95% confidence intervals (95% CI) per 1 mmol/L increment in glucose level for AKI were calculated by logistic regression. The receiver operating characteristic analysis was conducted and Youden’s index which maximizes the sum of sensitivity and specificity was used to identify the cut-off value for preoperative glucose to predict AKI. Wald *χ*2 test was used to evaluate the difference between the two cohorts, The adjusted OR and 95% CI comparing both cohorts with DM and without DM on AKI over the range of preoperative glucose values from 3 to 14 mmol/L were estimated.

**Table 1:** The characteristics of patients undergoing non-cardiac surgery according to diabetic status

Variables	All patients (*n* = 39986)	DM (*n* = 2375)	Non-DM (*n *= 37611)
Age	52(44,61)	62(54,69)	51(44,60)
Gender			
Male	12473(31.2%)	1020(42.9%)	11437(30.4%)
Female	27513(68.8%)	1355(57.1%)	26174(69.6%)
BMI	23.4(21.4,25.7)	24.3(22.4,26.7)	23.4(21.3,25.6)
Co-morbidity			
Stroke	1001(2.5%)	171(7.2%)	824(2.2%)
Hypertension	6298(15.8%)	1202(50.6%)	5080(13.5%)
CAD	873(2.2%)	209(8.8%)	663(1.8%)
Arrhythmia	2147(5.4%)	132(5.6%)	2012(5.3%)
DM	2381(6.0%)	/	/
COPD	159(0.4%)	19(0.8%)	139(0.4%)
Asthma	211(0.5%)	11(0.5%)	199(0.5%)
Renal disease	120(0.3%)	27(1.1%)	91(0.2%)
Liver disease	905(2.3%)	67(2.8%)	839(2.2%)
Thyroid diasese	501(1.3%)	30(1.3%)	473(1.3%)
Cancer	741(1.9%)	46(1.9%)	690(1.8%)
Smoker	4767(11.9%)	338(14.2%)	4426(11.8%)
Preoperative medication			
Antihypertension	1539(3.8%)	309(13.0%)	1230(3.3%)
Antiplatelets	297(0.7%)	77(3.2%)	220(0.6%)
Hypoglycemic	1413(3.5%)	1323(55.7%)	90(0.2%)
Anticoagulants	52(0.1%)	3(0.1%)	49(0.1%)
Thyroxine	223(0.6%)	16(0.7%)	207(0.6%)
Methimazole	51(0.1%)	2(0.1%)	49(0.1%)
Preoperative blood test			
Hb(g/L)	127(112,138)	126(108,138)	127(112,138)
Glucose(mmol/L)	4.81(4.37,5.34)	6.73(5.46,8.39)	4.77(4.35,5.25)
Albumin(g/L)	42.3(39.0,45.5)	41.2(38,44.6)	42.4(39.1,45.6)
Cr(umol/L)	55.7(48.4,66.2)	58.1(47.8,70.7)	55.6(48.4,65.9)
Intraoperative period			
Surgical duration(min)	111(65,183)	135(81,215)	110(65,180)
Fluid infusion rate (ml/kg/h)	11.2(8.3,15.4)	10.1(7.7,13.4)	11.3(8.4,15.5)
Bleeding(ml)	90(20,100)	100(30,100)	80(20,100)
Transfusion	1182(3.0%)	103(4.3%)	1075(2.9%)
NA infusion	10332(25.8%)	947(39.9%)	9359(24.9%)
NSAIDs	1889(4.7%)	80(3.4%)	1809(4.8%)
Any hypotension(Y vs N)	14949(37.4%)	839(35.3%)	14131(37.6%)
Colloid infusion (Y vs N)	12764(31.9%)	942(39.7%)	11822(31.4%)
Body temperature (^°^C)	36.3(36.2,36.5)	36.4(36.2,36.5)	36.3(36.2,36.5)
AKI *n*(%)	741(1.9%)	134(5.6%)	607(1.6%)

*CAD* coronary artery disease, *DM* diabetes mellitus, *COPD* chronic obstructive pulmonary disease, *Hb* hemoglobin, *Cr* creatinine, *NA* noradrenaline, *NSAIDs* non-steroid anti-inflammatory drugs, *Y vs N* yes vs no, *AKI* acute kidney injury

The data were analyzed using Stata16 (Stata Corp.) and R software 4.12(R foundation for statistical computing). The significance of all tests was two-sided, and *P* < 0.05 was considered statistically significant.

## Results

**Fig. 1:**
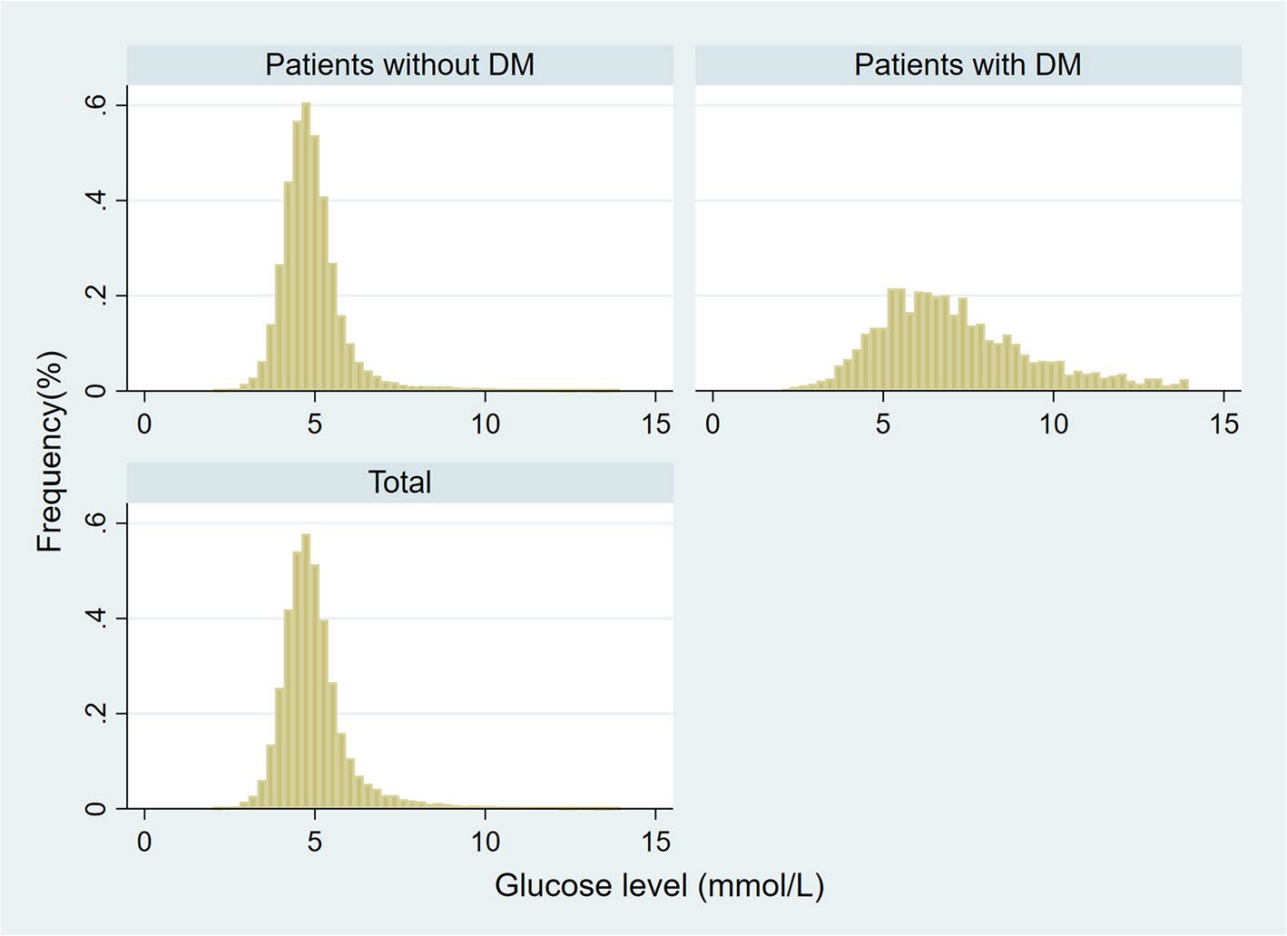
The distribution of preoperative glucose values in patients with and without diabetes in 1 mmol/L increment

Data from 44,541 patients undergoing elective non-cardiac surgery in our institution between January 2018 and May 2023 were available. The excluded were patients with missing preoperative blood glucose values (*n* = 3650), patients with missing intraoperative blood pressure values (*n* = 560), and patients with missing postoperative creatinine values (*n* = 345), and finally, 39,986 patients were included for data analysis. The frequency histograms are shown in Fig. 1. The characteristics of All patients, patients with DM, and patients without DM are presented in Table 1. The median (interquartile range) glucose values in DM cohort and non-DM cohort were 6.73(5.46, 8.39) mmol/L and 4.77(4.35, 5.25) mmol/L, 741(1.9%) patients developed AKI, 134(5.6%) in patients with DM, and 607(1.6%) in patients without DM (OR 1.312, 95% CI 1.028–1.675, *P* = 0.029).

**Fig. 2:**
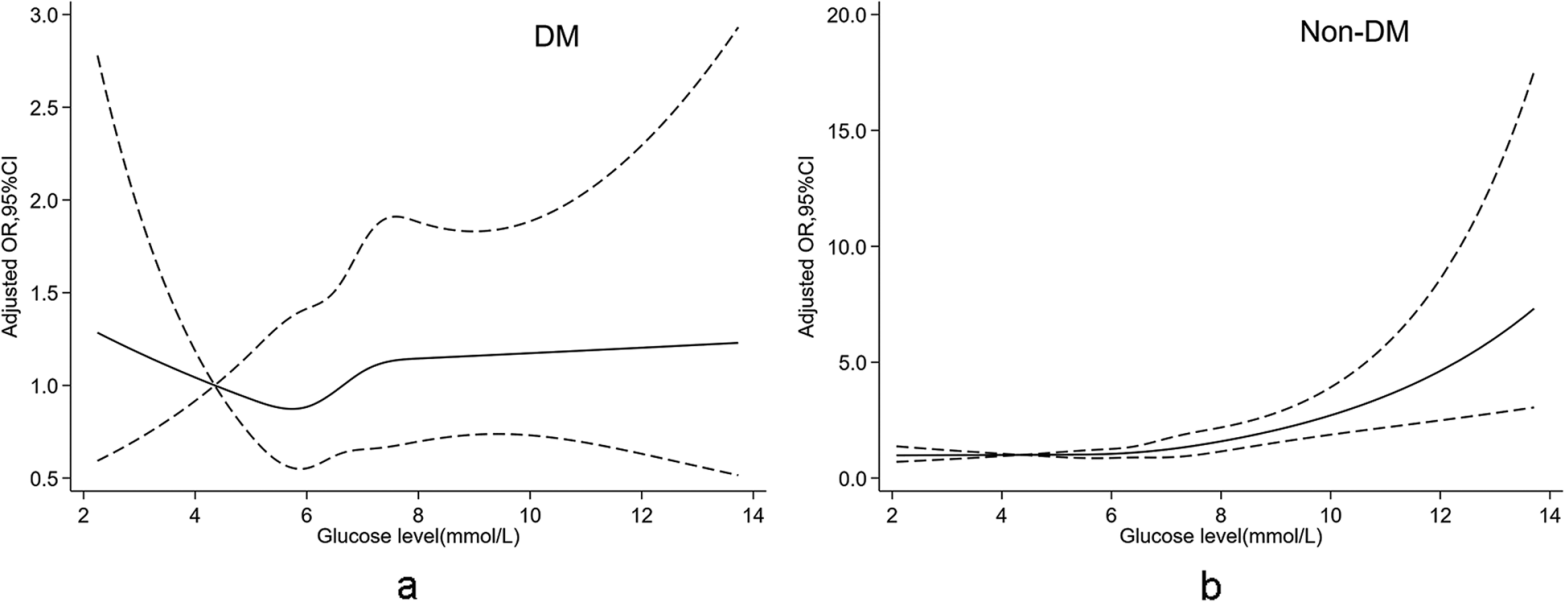
The adjusted ORs for AKI according to preoperative glucose values are based on the restricted cubic spline model. The solid black lines represented ORs adjusted for all variables listed in Table 1, and the dashed lines represented the upper and lower 95% CI

A significant non-linear association between preoperative glucose and postoperative AKI exists in patients without after covariable adjustment (*P* = 0.000, Fig. 2). The preoperative glucose level was progressively related to a higher risk of AKI, and every 1 mmol/L increment of preoperative glucose level increased the adjusted OR for AKI by 15% (adjusted OR 1.150, 95% CI 1.078–1.227, *P* = 0.000). But in patients with DM, the relationship between preoperative glucose and postoperative AKI was not significant after adjusting for covariables (*P* = 0.437, Fig. 2), the risk of AKI was at the lowest level at 5.80 mmol/L of glucose value, and when it is between 4.37 mmol/L and 6.70 mmol/L, the OR was below 1. Compared with this range, the adjusted OR for AKI at the preoperative glucose value below 4.37 mmol/L was 0.849 (95% CI 0.417–1.726, *P* = 0.650), and the adjusted OR of AKI at the preoperative glucose value above 6.70 mmol/L was 1.212 (95%CI 0.808–1.816, *P* = 0.353). The relationship between preoperative blood glucose and postoperative AKI was significantly different between patients with and without DM when compared using the Wald *χ*2 test (*P* = 0.000).

According to the Youden’s Index from the receiver operating characteristic analysis, the optimal cut-point of preoperative fasting glucose level to predict AKI was 5.39 mmol/L (adjusted OR 1.802, 95%CI 1.513–2.146, *P* = 0.000) in patients without DM, and the AUC was 0.577 (Supplementary Figure S1).

**Fig. 3:**
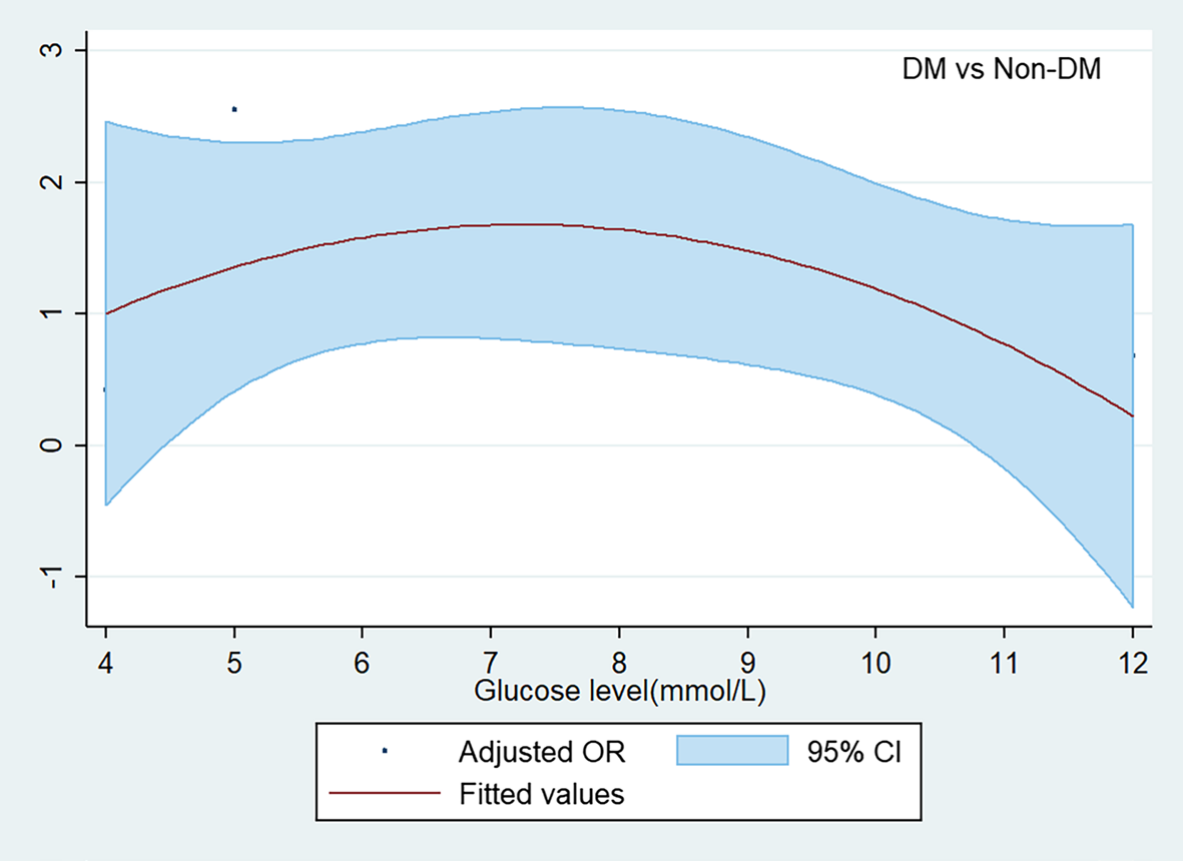
The adjusted OR for AKI in patients with DM versus without DM. The solid black lines represented ORs adjusted for all variables listed in Table 1, and the dashed lines represented the upper and lower 95% CI

The curve of the adjusted OR comparing patients with and without DM on AKI was shown in Fig. 3, and it was significantly and non-linearly associated with preoperative glucose values (*P* = 0.000). The odds of AKI were increased with the progression of preoperative glucose level when it was below 7.35 mmol/L and then decreased with the progression of its value when it was above 7.35 mmol/L, no significance existed between both cohorts over the range of preoperative glucose from 4 to 12 mmol/L.

## Discussion

In our study, the main findings were as follows: the preoperative glucose value was significantly associated with postoperative AKI in patients without DM, and its value of 5.39 mmol/L could predict AKI in non-cardiac surgery, the relation was not significant in patients with DM. Compared with patients without DM, the risk for AKI was not different from those with DM over the range of preoperative glucose. These results indicated that tight control of preoperative glucose might be necessary to reduce postoperative AKI in patients without DM undergoing non-cardiac surgery.

Preoperative hyperglycemia is associated with an increased risk of postoperative complications (Davis et al. 2012; Mao et al. 2020; Noordzij et al. 2007). Hyperglycemia can often occur before surgery in diabetic patients(glycemic poor control) and non-diabetic patients(unrecognized DM or stress). As long-term hyperglycemia in DM can cause chronic kidney injury (Lytvyn et al. 2020), the impact of transient hyperglycemia before surgery on AKI is not clear. This study showed that the preoperative glucose level was progressively related to a higher risk of AKI in patients with non-DM, but in patients with DM, the preoperative glucose level was not related to AKI. In one retrospective cohort study, Abdelmalak et al. reported no significant relationship between glucose level and 1-year mortality after non-cardiac surgery for patients with diagnosed diabetes, but a strong relationship among patients without diabetes (Abdelalak et al. 2014). In another prospective cohort study, preoperative fasting glucose was not associated with myocardial injury after non-cardiac surgery in patients with diabetes, but in patients without diabetes (Punthakee et al. 2018). These results were consistent with our results, and the relation between preoperative fasting glucose and postoperative complications in diabetic patients needs to be elucidated in large sample-size randomized controlled trials.

Till now, no consensus on the controlled target of preoperative or perioperative glucose level has been reached, some guidelines recommend perioperative glucose value below 10 mmol/L or between 6 and 10 mmol/L to avoid hyperglycemia or hypoglycemia (Dhatariya et al. 2012; Cosson et al. 2018; Association of Anaesthetists of Great Britain and Ireland 2015), but the European Society of Anesthesiology guideline recommend no routine preoperative glucose assessment for non-cardiac surgery (Hert et al. 2018). Many studies reported the threshold values of preoperative glucose for a specific complication after surgery, which showed that the preoperative fasting glucose level of 5.135 mmol/L is the cut-off value to predict mortality within 30 days after neurosurgery (Zhang et al. 2023), and the level greater than 7 mmol/L can predict the development of stroke within 30 days after non-cardiac surgery (Liu et al. 2022); in another retrospective study, the preoperative casual blood glucose value of 6.86 mmol/L in non-diabetic patients and 7.92 mmol/L in diabetic patients could predict postoperative myocardial injury in non-cardiac surgery (Punthakee et al. 2018). In our study, the cut-off value of preoperative fasting glucose was 5.39 mmol/L to predict AKI in non-cardiac surgery. These results indicated that the cut-off values were different regarding each specific complication.

There are several mechanisms for how preoperative hyperglycemia can cause postoperative AKI. The animal studies showed that hyperglycemia could cause increased oxidative stress through the xanthine pathway, and cause obvious tubular morphological and functional injuries in rats (Hikrose et al. 2008; Vanhorebeek et al. 2009). Clinical evidence revealed that acute hyperglycemia led to increased urinary excretion of inflammatory cytokines/chemokines in humans (Cherney et al. 2011). In addition, hyperglycemia can lead to renal hypoxia, triggering a fibrotic response that causes loss of peritubular capillaries and ischemic injury (Basile et al. 2001), and providing a signal for activation of inflammatory cells (Kong et al. 2004), and precipitating the expression of hypoxia-inducible factor 1 α which is correlated with glomerulosclerosis (Eckardt et al. 2007).

The odds ratio for AKI was decreased from the comparison between patients with DM and without DM in our study when the preoperative glucose value was above 7.35 mmol/L, but this did not mean that DM was associated with better outcomes than non-DM when hyperglycemia occurred before surgery, as DM was associated with many other adverse outcomes. There are many confounding factors associated with AKI from multivariable regression analysis in our study (Supplementary Table S1), and preoperative hyperglycemia is a modifiable factor and can be a screening tool to predict AKI. Results from patients undergoing cardiac surgery or critically ill patients in the ICU showed that tight control of perioperative glucose could reduce the risk of AKI compared with conventional control (Krinsley 2004; Lecomte et al. 2008). The findings from our study raise the question of whether tight control of preoperative glucose could reduce AKI in non-cardiac surgery, to answer this question, a further randomized controlled trial is needed.

There are some limitations in our study. First, we only investigated the relationship between preoperative glucose and postoperative AKI, and the association was significant in non-DM patients. As postoperative glucose value is a very important risk factor for postoperative complications, we did not exclude the effect of postoperative glucose it. Second, preoperative hyperglycemia might be a short-term or long-term comorbidity, which could not be determined and might exert a different impact on postoperative AKI. Third, the relation was not significant between preoperative glucose and postoperative AKI in patients with DM probably due to the relatively small sample size, and we could not figure out the cut-off value to predict AKI in patients with DM. Fourth, our study was a retrospective cohort study, and a further randomized controlled trial is needed to verify the results in the near future.

## Conclusion

Preoperative fasting glucose value can predict the risk of postoperative acute kidney injury after non-cardiac surgery in patients without diabetes, and tight control of preoperative glucose might be necessary; but in patients with diabetes, the association was not significant. Large sample-size randomized controlled trials are needed to verify the results.

### Supplementary Information

Additional file 1: Supplemental Figure S1. The receiver operating characteristic curve for preoperative glucose. The AUC was 0.577.

Additional file 2: Supplemental Table S1. The independent risk factors from multivariate regression analysis.

## Data Availability

All the data can be obtained by contacting the corresponding author.
