# Supplementary figures and images for: Preoperative fasting glucose value can predict acute kidney injury in non-cardiac surgical patients without diabetes but not in patients with diabetes
Source: Perioper Med (Lond). 2024 May 13;13:39. doi: 10.1186/s13741-024-00398-4 (PMC11089748; doi:10.1186/s13741-024-00398-4)

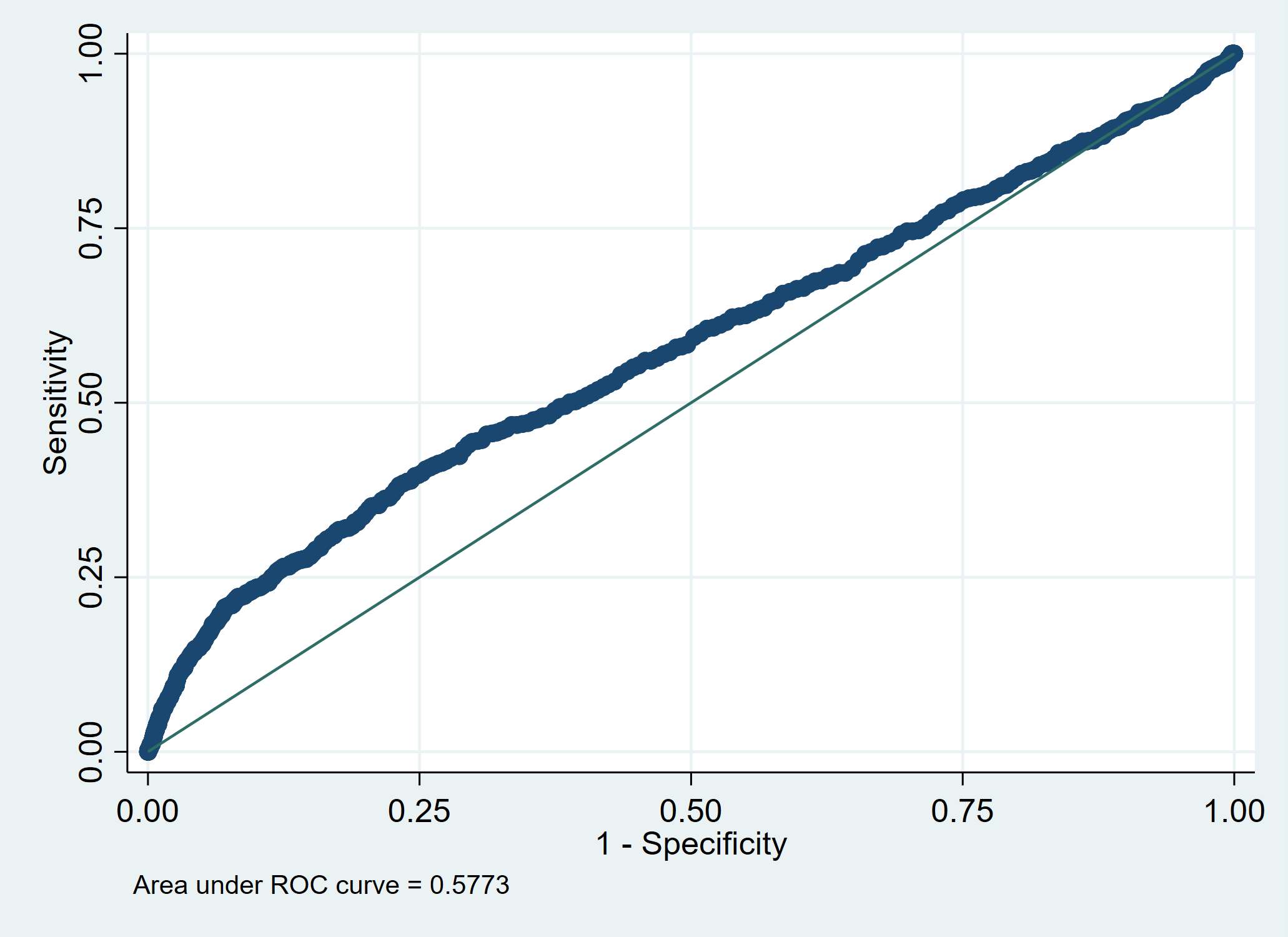

Supplement: Supplementary file 1 — Additional file 1: Supplemental Figure S1. The receiver operating characteristic curve for preoperative glucose. The AUC was 0.577. [file 13741_2024_398_MOESM1_ESM.png]
