# Supplementary material for: Preoperative fasting glucose value can predict acute kidney injury in non-cardiac surgical patients without diabetes but not in patients with diabetes
Source: Perioper Med (Lond). 2024 May 13;13:39. doi: 10.1186/s13741-024-00398-4 (PMC11089748; doi:10.1186/s13741-024-00398-4)
Supplement: Supplementary file 2 — Additional file 2: Supplemental Table S1. The independent risk factors from multivariate regression analysis. [file 13741_2024_398_MOESM2_ESM.docx]

Supplemental Table. The independent risk factors from multivariate regression analysis

| Variable | Adjusted OR,95%CI | *P* value |
| --- | --- | --- |
| Glucose (mmol/L) | 1.109(1.055,1.167) | 0.000 |
| Age(y) | 1.024(1.017,1.032) | 0.000 |
| Gender(female as reference) | 1.576(1.311,1.895) | 0.000 |
| Hypertension | 1.808(1.514,2.159) | 0.000 |
| Cardiac artery disease | 1.370(1.009,1.860) | 0.044 |
| Renal disease | 2.340(1.291,4.243) | 0.005 |
| Diabetes mellitus | 1.301(1.020,1.661) | 0.034 |
| Albumin(g/L) | 0.956(0.941,0.972) | 0.000 |
| Creatinine(umol/L) | 1.012(1.009,1.015) | 0.000 |
| Blood loss(ml) | 1.000(1.000,1.000) | 0.001 |
| Surgical duration(min) | 1.002(1.002,1.003) | 0.000 |
| Intraoperative hypotension | 1.346(1.148,1.579) | 0.000 |
| Intraoperative transfusion | 1.637(1.245,2.152) | 0.000 |
| Intraoperative NA infusion | 2.370(1.966,2.856) | 0.000 |
| Fluid infusion rate(ml/kg/h) | 1.013(1.000,1.025) | 0.044 |
